# Clindamycin-Loaded Polyhydroxyalkanoate Nanoparticles for the Treatment of Methicillin-Resistant *Staphylococcus aureus-*Infected Wounds

**DOI:** 10.3390/pharmaceutics16101315

**Published:** 2024-10-10

**Authors:** Muneeb Ullah, Juho Lee, Nurhasni Hasan, Md. Lukman Hakim, Dongmin Kwak, Hyunwoo Kim, Eunhye Lee, Jeesoo Ahn, Bora Mun, Eun Hee Lee, Yunjin Jung, Jin-Wook Yoo

**Affiliations:** 1 College of Pharmacy, Pusan National University, Busandaehak-ro 63 beon-gil 2, Geoumjeong-gu, Busan 46241, Republic of Korea; munibdawar72@gmail.com (M.U.); jhlee2350@gmail.com (J.L.); mlhshohag1221@gmail.com (M.L.H.); pharm0701@gmail.com (D.K.); rlagusdn0628@naver.com (H.K.); jungy@pusan.ac.kr (Y.J.); 2 Faculty of Pharmacy, Hasanuddin University, Jl. Perintis Kemerdekaan KM 10, Makassar 90245, Indonesia; nurhasni.hasan@unhas.ac.id; 3 CJ CheilJedang Corporation, Suwon 16495, Republic of Korea; eunhye.lee@cj.net (E.L.); jisoo.ahn@cj.net (J.A.); bora.mun@cj.net (B.M.); 4 College of Pharmacy, Korea University, Sejong 30019, Republic of Korea; eunheelee@korea.ac.kr

**Keywords:** polyhydroxyalkanoates, clindamycin, nanoparticles, MRSA-infected cutaneous wounds

## Abstract

**Background/Objectives:** Owing to the growing resistance of methicillin-resistant *Staphylococcus aureus* (MRSA) to conventional antibiotics, the development of innovative therapeutic strategies for the treatment of MRSA-infected cutaneous wounds poses a significant challenge. **Methods:** Here, by using polyhydroxyalkanoates (PHA), emerging biodegradable and biocompatible polymers naturally produced by various microorganisms, we developed clindamycin-loaded PHA nanoparticles (Cly-PHA NPs) as a novel approach for the treatment of MRSA-infected cutaneous wounds. **Results:** Cly-PHA NPs were characterized in terms of mean particle size (216.2 ± 38.9 nm), polydispersity index (0.093 ± 0.03), zeta potential (11.3 ± 0.5 mV), and drug loading (6.76 ± 0.19%). Owing to the sustained release of clindamycin over 2 days provided by the PHA, Cly-PHA NPs exhibited potent antibacterial effects against MRSA. Furthermore, Cly-PHA NPs significantly facilitated wound healing in a mouse model of MRSA-infected full-thickness wounds by effectively eradicating MRSA from the wound bed. **Conclusions:** Therefore, our results suggest that Cly-PHA NPs offer a promising approach for combating MRSA infections and accelerating cutaneous wound healing.

## 1. Introduction

A wound is defined as a disruption of the skin resulting in the deterioration of skin integrity, which provides a moist, warm, and nourishing condition for the colonization and growth of microorganisms that cause infection [1]. Bacterial infections account for a significant proportion of morbidity and death rates across the world [2]. *Staphylococcus aureus*, which includes methicillin-resistant *S. aureus* (MRSA), is the most common bacteria responsible for wound infections in healthcare settings [1,3]. MRSA is a significant nosocomial pathogen on a global scale, accounting for ≥40% and 25–30% of hospital-acquired *S. aureus* infections in large tertiary care institutions and smaller sites, respectively. The prevalence of MRSA infection has continued to rise, becoming a matter of public health concern [4]. Prolonged and extensive use of antibiotics has resulted in the development of bacteria resistant to antibiotics [1]. Hence, novel strategies must be devised for the treatment of MRSA-infected cutaneous wounds.

Topical administration of antibiotic-incorporated nanoparticles (NPs) for the treatment of MRSA-infected wounds has garnered the attention of researchers in recent years. Various kinds of antibiotic-loaded NPs have demonstrated enhanced antibacterial effects [5,6]. Furthermore, compared with conventional formulations, the use of NPs as a drug delivery system for the treatment of infected cutaneous wounds has exhibited various advantages [6,7]. The integration of antibiotics into NPs augments the efficacy of antibiotics, mitigates the incidence of side effects, decreases the likelihood of developing antibiotic resistance, and preserves elevated levels of the drug in the wound bed [8,9]. Nevertheless, the clinical application of antibiotic-loaded NPs remains underexplored due to challenges such as high cost, scalability, and ensuring consistent quality in large-scale production [10,11,12]. Although formulations composed of synthetic polymers like poly (lactic-co-glycolic acid) (PLGA) have demonstrated enhanced therapeutic effects, they are expensive and difficult to produce on a large scale, which hinders clinical translation [13,14,15]. Therefore, antibiotic-loaded NPs comprising cost-effective polymers must be developed.

Polyhydroxyalkanoates (PHAs), synthesized by a wide variety of microorganisms, are promising alternatives to conventional synthetic polymers [16]. PHA has been emerging as a novel biopolymer since PHA possesses excellent biocompatibility, biodegradability, adaptability, and high drug-loading capacity [17]. Compared to commonly used polyester materials like polylactic acid (PLA) and PLGA, which are Food and Drug Administration-approved implant materials, PHA presents distinct advantages. For instance, PLA degrades rapidly and releases lactic acid, which can cause non-bacterial inflammation in the implanted microenvironment, whereas PHA degradation products are safe and non-toxic [18]. Additionally, PHA offers long-term drug release compared to PLA and PLGA, making it highly suitable for drug delivery applications [19,20]. Furthermore, PHA is capable of releasing antibiotics in a regulated and sustained manner [21,22]. These properties render PHA suitable for incorporation into biomedical applications, particularly in drug delivery systems [8,9,16,23]. In particular, PHA offers significant advantages, as it can be produced from microorganisms, waste materials, and plant-based carbohydrates, significantly reducing production costs [24,25]. Unlike synthetic polymers, which require complex and expensive production processes, PHA can be manufactured in a more straightforward and scalable manner, making it a practical option for large-scale pharmaceutical applications [26,27]. Its ability to be produced in high volumes at lower costs further enhances its appeal for clinical and industrial use, where affordability is crucial for widespread adoption. For these reasons, PHA has gained considerable attention for its therapeutic applications as a delivery carrier for antibacterial [28,29], antifungal [30], and anticancer drugs [31].

In this study, we propose the novel application of PHAs as antibiotics-loaded NPs for the treatment of MRSA-infected cutaneous wounds. By using clindamycin as a model drug, clindamycin-loaded PHA NPs (Cly-PHA NPs) were successfully developed. After physicochemical characterizations of Cly-PHA-NPs, the in vitro antibacterial effects of Cly-PHA NPs were investigated against MRSA. Furthermore, the in vivo wound-healing effects of Cly-PHA NPs were evaluated using an MRSA-infected full-thickness wound mouse model.

## 2. Materials and Methods

### 2.1. Materials

3-hydroxybutyrate-co-4-hydroxybutyrates (short-chain PHA copolymers, produced by genetically modified *Escherichia. coli* K-12 strains, Mw: 700 kDa) were kindly donated by CJ Cheiljedang (Suwon, Republic of Korea). Clindamycin HCl was a generous gift from the Samjin Pharmaceutical Company (Seoul, Republic of Korea). Bacto^TM^ tryptic soy broth (TSB) was purchased from BD Biosciences and Life Technologies (San Jose, CA, USA). LIVE/DEAD^®^ Baclight^TM^ kit was purchased from Thermo Fisher Scientific (Waltham, MA, USA). Polyvinyl alcohol (PVA), 2,2,2-tribromethanol, and tert-amyl alcohol were purchased from Sigma-Aldrich (St. Louis, MO, USA). Masson’s trichrome staining kit was purchased from Abcam (Cambridge, MA, USA). Twort’s Gram staining kit was purchased from Newcomer Supply (Middleton, WI, USA). All other solvents and reagents used in this study were of the highest analytical grade.

### 2.2. Preparation of Clindamycin-Loaded PHA NPs

Prior to the fabrication of Cly-PHA NPs, clindamycin base was extracted using a desalting method, as described in a previously reported method [32]. Cly-PHA NPs were prepared using the oil-in-water (*o*/*w*) emulsification solvent evaporation method with slight modifications [33]. In brief, 5 mg of clindamycin base and 50 mg of PHA polymer (1:10 ratio) were dissolved in 5 mL of chloroform through vortexing, using a 2% polyvinyl alcohol (PVA) solution as the emulsifier. The organic phase was added dropwise to 20 mL of an aqueous solution containing 2% PVA, and the mixture was stirred continuously at 500 revolutions per minute (rpm) to prepare a coarse emulsion. The mixture was further emulsified using a probe sonicator at 50% (150 W) for 3 min in an ice bath and transferred to 20 mL of a solution containing 0.5% PVA. The mixture was stirred at 500 rpm in a fume hood for 4 h to facilitate complete solvent evaporation. The NPs were collected by centrifugation at 20,000× *g* for 30 min. The NPs were washed twice using cold distilled water (DW) and stored at 4 °C in a refrigerator for further use.

### 2.3. Characterization of Cly-PHA NPs

Scanning electron microscopy (SEM; Supra 40VP, Main Central Facilities Lab, Pusan National University, Busan, Republic of Korea) was performed to observe the structural morphology of PHA NPs and Cly-PHA NPs. The samples were prepared by mounting the NPs on a carbon tape and subjecting them to platinum sputtering for 2 min under vacuum. The morphology of the NPs was observed within a size range of 200–300 nm at an acceleration voltage of 1–6 KV. ImageJ software (version 1.52i, National Institutes of Health, Bethesda, MA, USA) was used to measure the size of the NPs. Zetasizer Nano ZSS90 (Malvern Instruments, Malvern, UK) was used to measure the size and zeta potential of the NPs. High-performance liquid chromatography (HPLC; Shimadzu, Kyoto, Japan) was performed according to a previously reported method with slight modifications [21] to determine the concentration of clindamycin loaded into the PHA NPs. The HPLC system was equipped with the following: SPD-20A Prominence UV/Vis detector, LC-20AT liquid chromatograph, CT-20A Prominence column oven, DGU-20ASR degassing unit, and SIL-20 Prominence autosampler. VDSpher^®^ PUR 100 C18-M-SE columns (5 µm, 4.6 × 250 mm, VDS Opti lab, Berlin, Germany) were used. A standard stock solution of clindamycin was prepared by dissolving clindamycin in a mobile phase comprising acetonitrile and potassium phosphate buffer (pH 3.0) (50:50 *v*/*v*). The sample was sonicated, filtered, and centrifuged to prevent column blockage, and pumped at a flow rate of 500 μL/min. This sample (10 µL) was injected and evaluation was performed at 210 nm. A certain amount of Cly-PHA NPs was dissolved in chloroform, sonicated for 4 h, and centrifuged at 17,000× *g* for 30 min. The supernatant was diluted with the mobile phase to a suitable dilution, and 200 µL of each sample was transferred to the HPLC autosampler. HPLC was performed under the same chromatographic conditions as described previously. The area under the curve of the clindamycin concentration was linearly correlated (R^2^ = 0.998) within a concentration range of 0.00048–1 mg/mL and performed in triplicate.

### 2.4. In Vitro Drug Release Study

The in vitro release of clindamycin in phosphate buffer saline (PBS; pH 7.4 [physiological pH]) at 37 °C was evaluated. Cly-PHA NPs (2 mg) were suspended in 2 mL of the release medium and prepared in triplicate at each time interval. The samples were shaken at 120 rpm in a shaking incubator. The samples were collected at predetermined time intervals and centrifuged at 17,000 rpm for 30 min. The supernatant was diluted with the mobile phase. Tween-80 (2% *w*/*v*) was added to the release media to enhance the solubility of Cly-PHA NPs and facilitate the release of clindamycin. Approximately 200 µL of the sample was taken at predetermined time periods (1, 2, 4, 6, 12, 24, 36, and 48 h) and transferred to the HPLC autosampler. The supernatant containing clindamycin was analyzed using Shimadzu HPLC as described in the protocols mentioned previously. The procedure was performed in triplicate.

### 2.5. Physical Stability of NPs

The PHA NPs and clindamycin-loaded PHA NPs (Cly-PHA NPs) were prepared as described in Section 2.2 and stored at 4 °C for further use. The size and zeta potential were assessed daily for one week using a zetasizer. Before each measurement, the NPs were dispersed, and their size and zeta potential were analyzed.

### 2.6. In Vitro Antibacterial Study

The MRSA bacterial strain (USA-300) was used to evaluate the antibacterial activity of Cly-PHA NPs. The bacterial colony-forming units (CFU) were counted and a live/dead assay was performed using confocal microscopy to determine the antibacterial activity of the NPs [34]. The bacterial strain was inoculated in TSB and cultured to the exponential phase at 37 °C in a shaking incubator. The bacterial suspension was centrifuged at 6000× *g* for 6 min, washed twice with sterile PBS, and adjusted to an optical density of 0.2 at 600 nm. TSB was added to the adjusted suspension. The NPs suspension (PHA NPs and Cly-PHA NPs) was added subsequently to achieve final concentrations of 1, 2, 4, and 6 mg/mL. The samples were incubated at 37 °C for 8, 16, and 24 h. The bacterial suspensions were diluted in PBS to a concentration of 108 CFU/mL to determine bacterial viability by counting the number of CFUs. Following dilution, 100 µL of each dilution was spread on TSB agar plates and incubated overnight at 37 °C. The number of bacterial colonies was counted to determine the number of viable bacteria. The reagents of the LIVE/DEAD^®^ Baclight^TM^ bacterial viability kit was used in accordance with the manufacturer’s instructions to determine the number of live and dead bacteria. Confocal microscopy was performed at the Department of Pharmacy, Pusan National University, to capture bacterial images. Live bacteria were stained with CYTO-9 (green) at an excitation/emission (ex/em) wavelength of 539/570–620 nm, whereas dead bacteria were stained with propidium iodide (PI) (red) at an excitation/emission (ex/em) wavelength of 470/490–540 nm.

### 2.7. In Vivo Wound Healing Assay

The animal experiments conducted in this study adhered to the regulations and were approved by the Ethical Committee of Pusan National University, Institutional Animal Care Unit and Use Committee (PNU-IACUC), Busan, Republic of Korea (registration number: PNU-2023-0090; March 2023). Six-week-old male ICR mice were used to create the MRSA-infected cutaneous wound model. The mice were first anesthetized with avertin, and the dorsal area was shaved with an electric trimmer. An 8-mm full-thickness wound was created using an 8-mm biopsy punch. MRSA infection was induced by applying a suspension of MRSA containing 1.0 × 10^9^ CFU to the wound site. Treatment with PHA NPs and Cly-PHA NPs at a concentration of 6 mg/mL was commenced on day 2 post-injury after confirming infection of the wound with MRSA. The mice were divided into three groups: the untreated, PHA NPs, and Cly NPs groups. The wound was covered with Tegaderm^®^ (3M Company, St. Paul, MN, USA) and surgical gauze after treatment. The untreated group served as the control group. The gauze and Tegaderm^®^ were replaced every 2 days. Photographs of the wounds were taken and analyzed using the ImageJ software to determine the reduction in wound size.

### 2.8. Histological Assay

The ICR mice were sacrificed after the wound-healing process was completed, and cross-sectional full-thickness skin samples were collected. The samples were fixed in formaldehyde for 24 h. The skin tissue was dehydrated using a series of ethanol concentrations and embedded in paraffin. Vertical sections of 5 µm thickness were mounted on adhesive slide glasses. These sections were stained with hematoxylin and eosin (H&E) and Masson’s trichrome (MT) in accordance with standard protocols to facilitate morphological observation. A digital camera (Olympus DP23) mounted on a compound microscope (Olympus, Tokyo, Japan) was used to acquire histological images at a resolution of 1360 × 1024 pixels.

### 2.9. Reduction of Bacterial Wound Burden

The bacterial burden at the wound site was monitored on day 2 and day 12 post-injury. A sterile PBS swab was applied at the site of MRSA inoculation on day 2 post-injury and spread onto TSB agar plates for quantitative examination and accurate counting of the CFUs present at the wound site. The wound tissue was vortexed in PBS and diluted on day 12 as described previously. After dilution, 100 µL of each dilution was spread on TSB agar plates and incubated overnight at 37 °C. The CFUs were counted to calculate the number of viable bacteria.

### 2.10. Statistical Analysis

All data are presented as the means ± SD. GraphPad Prism version 8.0 was used for multiple comparisons.

## 3. Results

### 3.1. Characteristics of the NPs

**Figure 1:**
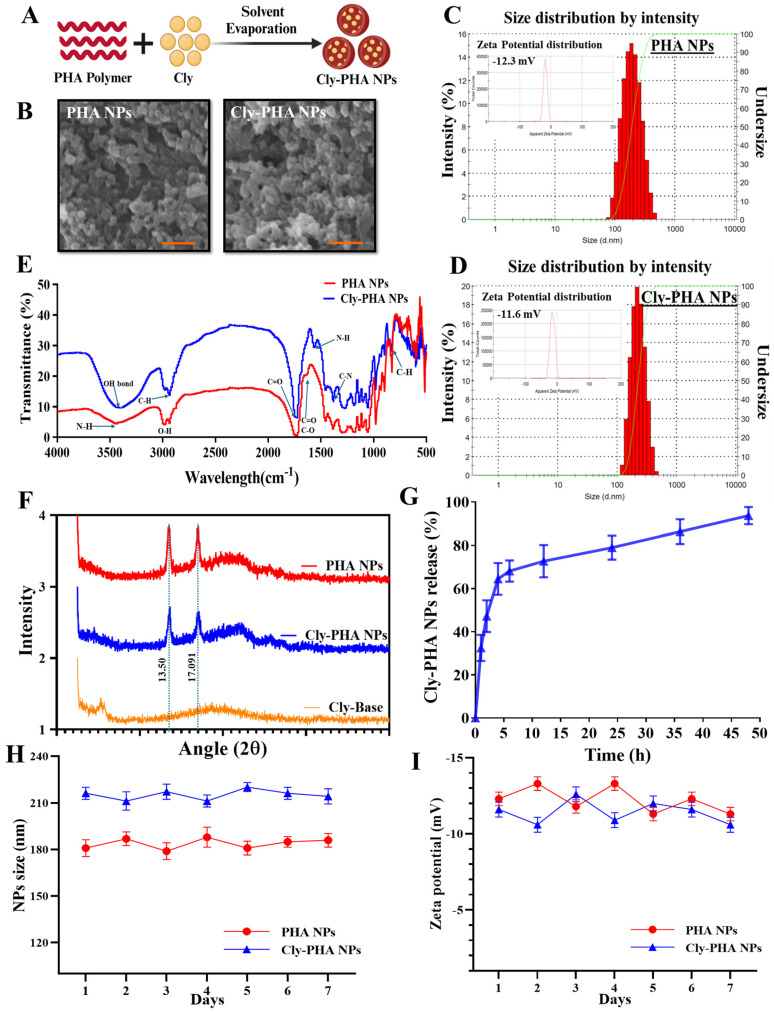
Characteristics of the nanoparticles (NPs): (**A**) schematic illustration of the synthesis of NPs; (**B**) scanning electron microscopy (SEM) images of polyhydroxyalkanoate NPs and clindamycin-loaded polyhydroxyalkanoate NPs (PHA NPs and Cly-PHA NPs, respectively); (**C**,**D**) size distribution of PHA NPs and Cly-PHA NPs by zetasizer, with the inset images representing the size and zeta potential of the NPs; (**E**) FTIR spectra of PHA NPs and Cly-PHA NPs; (**F**) XRD spectra of PHA and Cly-PHA NPs; and (**G**) Release profile of clindamycin from PHA NPs (Cly-PHA NPs) at pH 7.4. (**H**) Represents the physical stability of NP size at 4 °C for one week, and (**I**) the zeta potential values of the NPs assessed daily.

All NPs were prepared using the oil-in-water (*o*/*w*) emulsion solvent evaporation technique (Figure 1A). Table 1 presents the physicochemical properties, including morphology, size, polydispersity index (PDI), zeta potential, drug loading, and encapsulation efficiency of the NPs. The SEM images revealed the spherical morphology of PHA NPs and Cly-PHA NPs (Figure 1B). The average sizes of PHA NPs and Cly-PHA NPs were approximately 180.9 ± 27.4 nm and 216.2 ± 38.9 nm, respectively, indicating moderate PDI values. The zeta potential of PHA NPs was −12.3 ± 0.4 mV, similar to that of Cly-PHA NPs (−11.6 ± 0.5 mV; Figure 1C,D). This finding indicates that loading the NPs with clindamycin had no effect on the zeta potential of the polymer. The clindamycin loading in the PHA NPs (drug loading [DL%]) was 6.76 ± 0.19, corresponding to an encapsulation efficiency (EE%) of 74.06 ± 2.41%.

The Fourier-transform infrared spectroscopy (FTIR) spectra of PHA NPs and Cly-PHA NPs were measured to observe distinctive peaks (Figure 1E). Stretching from C=O and C-O was observed and compared with that observed in previously reported PHA. The strong peaks at 1726.17 cm^−1^ correspond to the C=O stretch of the ester bond, whereas the peaks between 1683.24 cm^−1^ and 1578.72 cm^−1^ correspond to the C=O and N-H stretching groups, respectively (Figure 1E). The stretching peaks at 2962.46 cm^−1^ and 2877.60 cm^−1^ correspond to the C-H bond of clindamycin and O-H groups owing to PHA, respectively. The stretching peaks of PHA were similar to those observed previously, indicating the successful formation of PHA and Cly-PHA NPs [35,36]. The loading of clindamycin into the PHA NPs had no significant effect on FT-IR peaks. The peaks of Cly-PHA NPs observed at 3401 cm^−1^, corresponding to N-H stretching, and C-N at 1457 cm^−1^ and 788 cm^−1^, respectively, were similar to those reported by previous studies [37]. X-ray diffraction (XRD) revealed diffraction peaks at 2θ values of 13.50° and 17.091°, corresponding to the miller indices [38]. The high-intensity peaks corresponded to the neat PHA crystal, whereas the zigzag conformation suggested a decrease in crystallinity (Figure 1F). The addition of the clindamycin base to the PHA copolymer had no effect on the intensity of the PHA peaks, as described in a previous study [38].

### 3.2. In Vitro Drug Release

The release of clindamycin from the Cly-PHA NPs followed a biphasic trend (Figure 1G). Up to 68.0 ± 4.9% of clindamycin was released within the first 6 h, followed by a more sustained release over the next 6 to 48 h, resulting in a total release of up to 93.7 ± 4.0% from Cly-PHA NPs. The extended-release profile allows Cly-PHA NPs to provide antibiotic delivery for more than 2 days, reducing the need for frequent dosing, which can improve patient convenience. Moreover, when applied to infected wounds, bacteria could be consistently exposed to clindamycin for 2 days, leading to enhanced eradication of bacteria from the wound sites.

### 3.3. Assessment of Nanoparticle Stability

The PHA NPs and Cly-PHA NPs were stored at 4 °C, and their size and zeta potential were monitored to assess the stability of NPs, as shown in Figure 1H, I. No significant changes were observed during the storage period, with variations in NP size being less than 7%, especially in the cases of PHA NPs (<6.41%) and Cly-PHA NPs (<4.18%). Although slight fluctuations in zeta potential were noted, overall, the NPs maintained a negative charge (Figure 1I), ensuring that all the NPs remained physically stable under the storage conditions.

### 3.4. In Vitro Antibacterial Activity of NPs

**Figure 2:**
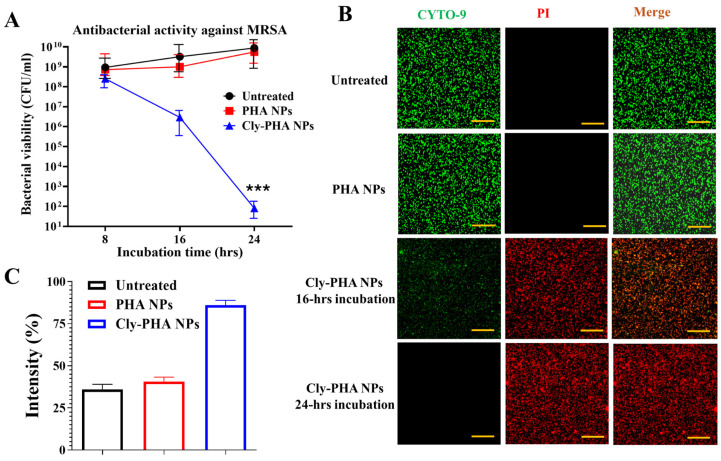
Antibacterial activity of polyhydroxyalkanoate nanoparticles and clindamycin-loaded polyhydroxyalkanoate nanoparticles (PHA NPs and Cly-PHA NPs, respectively) against methicillin-resistant *Staphylococcus aureus* (MRSA). (**A**) Antibacterial effects of PHA NPs and Cly-PHA NPs were evaluated at 8, 16, and 24 h using the plate counting method (*** indicates *p* < 0.001 compared to untreated and PHA NP-treated groups). (**B**) Representative fluorescence images of live/dead assay: CYTO-9 (green) staining represents live bacteria and PI (red) staining represents the dead bacteria. Confocal scale bar was set at 20 µm. (**C**) Fluorescence intensity of MRSA in the live/dead assay of the untreated, PHA NPs, and Cly-PHA NPs groups. The results are presented as means ± standard deviations (*n* = 3).

A CFU counting assay and a live/dead assay with confocal microscopy to visualize live and dead bacteria were performed to assess the antibacterial activity of PHA NPs and Cly PHA NPs against MRSA. The results presented in Figure 2A demonstrate the time-dependent effects of PHA and Cly-PHA NPs on MRSA. The untreated group was used as the control. PHA NPs exhibited no significant antibacterial effect following incubation at 37 °C for different intervals (8, 16, and 24 h). The Cly-PHA NPs exhibited no substantial antibacterial effect after 8 h of incubation. However, a 4-log and 7-log reduction in bacterial viability was observed after 16 h and 24 h of incubation (approximately 99.9% of bacterial killing), respectively, with a 6 mg/mL NP concentration. Figure 2B presents the antibacterial effect of PHA NPs and Cly PHA NPs, visualized using confocal microscopy after live/dead staining, compared with that in the untreated group (control group). CYTO-9 was used to stain live bacteria green, whereas PI was used to stain dead bacteria red. Uncountable live bacteria were visible in the images of the untreated group. PHA NPs also exhibited uncountable live bacteria, indicating no antibacterial effect even at high concentrations (6 mg/mL) after 24 h of incubation at 37 °C. In contrast, relatively few live bacteria were observed after 16 h of incubation with Cly-PHA NPs. Almost no live bacteria were observed when the incubation time was increased to 24 h (numerous dead bacteria were detected). The results were quantified based on the fluorescence intensities of live and dead bacteria (Figure 2C). This quantification confirmed that Cly-PHA NPs exerted a strong antibacterial effect, whereas PHA NPs failed to exhibit any antibacterial activity. The live/dead assay results were correlated with a 7-log reduction in bacterial viability (CFUs), indicating the death of approximately 99.9% of bacteria.

### 3.5. In Vivo Wound Healing Activity

**Figure 3:**
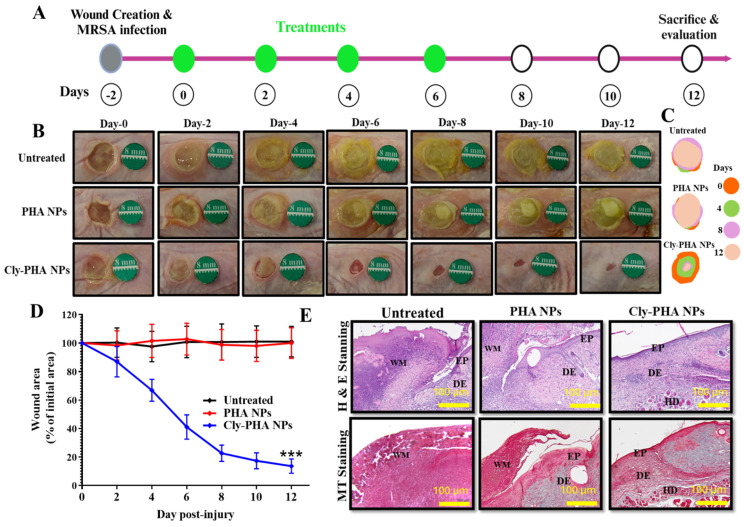
In vivo healing activity of polyhydroxyalkanoate nanoparticles and clindamycin-loaded polyhydroxyalkanoates nanoparticles (PHA NPs and Cly-PHA NPs, respectively) against methicillin-resistant *Staphylococcus aureus* (MRSA)-infected wounds. (**A**) Schematic illustration of the experimental flow. (**B**) Representative photographs of MRSA-infected wounds treated with PHA NPs and Cly-PHA NPs. (**C**) Schematic presentation of wound area on days 0, 4, 8, and 12 post-injuries. (**D**) Area reduction (%) profiles of the wound size (*** indicates *p* < 0.001 compared to the untreated and PHA NPs groups). (**E**) Histological analysis (hematoxylin and eosin and Masson’s trichrome staining) of the MRSA-infected wound on day 12, with the same scale bar used for normal wounds.

In vivo wound healing activity was evaluated to determine the healing efficacy of Cly PHA NPs in MRSA-infected wounds (Figure 3). The effects of PHA NPs and Cly PHA NPs were investigated and compared with those of the untreated group to ascertain the significant wound healing activity against MRSA-infected wounds (Figure 3). Similar to the untreated group, no significant effects were observed in the PHA NPs group. Clindamycin was loaded into the PHA NPs to disperse the bacterial burden at the wound site and facilitate wound healing. Figure 3A presents a schematic of the experimental flow. No significant effect on the bacterial burden at the wound site was observed in the PHA NPs group; in contrast, a significant reduction in the bacterial burden, followed by significantly faster wound healing, was observed in the Cly-PHA NPs group (Figure 3B). Figure 3C presents a schematic representation of the wound area on days 0, 4, 8, and 12. A reduction in the bacterial burden was observed on days 4 and 6 post-injury in the Cly-PHA NPs group. Wound size reduction was also determined to compare the healing effects observed in the untreated, PHA NPs, and Cly-PHA NPs groups. Approximately 100% ± 11.11 SD and 35.069 ± 8.5 SD, 22.72 ± 5.72 SD clindamycin was released in response to PHA NPs and Cly-PHA NPs on days 6 and 8, respectively (Figure 3D).

### 3.6. Histological Analysis

Histological examination of the wound site skin sample revealed skin morphology and collagen deposition after H&E and MT staining. Similar to the in vivo wound healing experiment, the mice were divided into three groups: the untreated, PHA NPs, and Cly-PHA NPs groups. The untreated and treated (PHA NPs and Cly-PHA NPs) mice exhibited abnormal skin morphology (Figure 3E). A lack of a skin layer (epidermal and hypodermal), as well as a lack of collagen deposition, was observed in the untreated group. However, the skin morphology was similar to that observed in the PHA NPs group, indicating that the PHA NPs had no significant wound healing activity. Fibroblast-like cells, formation of skin layers, and collagen deposition that closely resembled healthy skin morphology were observed in the Cly-PHA NPs group on day 12.

### 3.7. Wound Site Bacterial Burden Reduction

**Figure 4:**
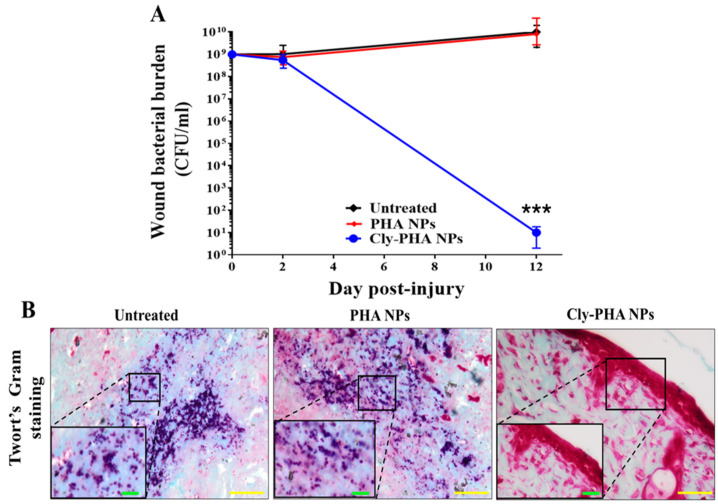
In vivo wound bacterial burden determined through the viable count of bacteria at the wound site. (**A**) Wound site bacterial burden log reduction through colony forming unit (CFU) counting assay. (**B**) Twort’s Gram staining images. Scale bar = 20 µm at 40× magnification, with an inset magnified to 100× showing a scale bar of 10 µm; *** indicates *p* < 0.001 (statistical significance compared with that in the untreated and PHA NPs groups).

Bacterial infections delay wound healing. Therefore, the MRSA bacterial burden at the wound site was evaluated to assess the antibacterial effects of Cly-PHA NPs compared with those of the PHA NPs and untreated groups. The untreated and PHA NPs groups exhibited no significant decrease in the number of MRSA bacterial colonies at the wound site on days 2 and 12 post-injury (Figure 4A). In contrast, the Cly-PHA NPs group exhibited a dramatic reduction in bacterial burden starting on day 2 post-injury, with an impressive bacterial reduction exceeding 98.8999% recorded on day 12. Swabs acquired from the wound site on days 2 and 12 revealed MRSA colonies in the untreated, PHA NPs, and Cly-PHA NPs groups. Twort’s Gram staining performed to visualize bacteria at the wound site revealed the presence of round-shaped dark blue dots, approximately 1–2 µm in size, representing Gram-positive MRSA (Figure 4B). These effects were observed in the untreated and PHA NPs groups. However, no dark blue dots were observed in the Cly-PHA NPs group, indicating that only a small number of bacteria could be detected through Twort’s Gram staining. The PHA NPs exerted no significant antibacterial effects compared with those observed in the untreated group. In contrast, no visible bacterial colonies were observed in the Cly-PHA NPs group, suggesting the complete eradication of the MRSA bacterial burden at the wound site.

## 4. Discussion

This study aimed to develop a clindamycin delivery system specifically targeting MRSA, thereby enhancing the effectiveness of antibiotics in treating MRSA-infected wounds. The loading of antibiotics into NPs has numerous advantages: (1) improved antibiotic effects and a reduction in the incidence of undesired side effects; (2) reduced risk of developing antibiotic resistance; (3) and high, sustained local drug concentration [39]. Antibiotics have been incorporated into polymeric NPs for the effective treatment of infective diseases in the past [40]. However, increasing the therapeutic efficacy with minimal side effects through the use of delivery systems, such as clindamycin delivery, is an ideal option for targeting pathogenic bacteria at the wound site. Characterization of PHA NPs and Cly PHA NPs revealed that the size and zeta potential of the NPs did not differ significantly, demonstrating that clindamycin loading had no effect on particle size and distribution. Thus, Cly-PHA NPs were successfully developed for the treatment of MRSA-infected wounds.

The in vitro clindamycin release from PHA NPs under physiological conditions in PBS (pH 7.4) at 37 °C was assessed before evaluating the antibacterial effect of Cly-PHA NPs. Clindamycin was released from the PHA NPs in a sustained manner, followed by a prolonged release for up to 48 h (Figure 1G). This release pattern explains the efficacy of clindamycin, which inhibited bacterial growth, followed by the bactericidal effect exerted by maintaining an adequate concentration of NPs at the site of action. Clindamycin is a bacteriostatic antibiotic that inhibits protein synthesis. The bactericidal effects against MRSA were achieved by increasing the concentration of clindamycin. Evaluating the antibacterial activity of PHA and Cly-PHA NPS against MRSA revealed that PHA NPs alone did not exhibit significant antibacterial activity; however, Cly-PHA NPs exhibited promising antibacterial activity against MRSA. Previous studies have validated the efficacy of antibiotic delivery through polymeric NPs, including clindamycin-loaded cationic poly (lactide-co-glycolic) acid (PLGA) NPs against MRSA-infected wounds [33], cationic hybrid (Eudragit/PLGA) NPs [41], antimicrobial peptides [42], chitosan NPs [43], and cationic peptide-based NPs [42].

Based on these results, Cly-PHA NPs can be considered effective against MRSA infection and capable of facilitating wound healing by eradicating the MRSA bacterial burden at the wound site. Full-thickness wounds in mice were inoculated with MRSA, and the MRSA-infected wounds were allowed to develop for 2 days. The tension around the wound was reduced owing to the elasticity of the back skin of the mice. Tegaderm^®^, a widely used adhesive film, was used to fix the skin around the wound to minimize this effect. The wounds were treated with NPs at 2-day intervals until day 8 based on the clindamycin release pattern from the PHA NPs. The bacterial burden at the wound site interrupted the wound-healing process by forming a barrier that was impenetrable to phagocytic cells and antibiotics, resulting in bacterial resistance. Thus, the eradication of the pathogenic bacterial burden from the wound bed is required to prevent severe local and systemic infections and facilitate wound healing [44].

The progress of wound healing was confirmed by conducting histological examination using H&E and MT staining of normal and MRSA-infected wounds. Figure 4 presents images of MRSA-infected wounds treated with PHA and Cly-PHA NPs. PHA NPs exhibited no significant antibacterial effect. In contrast, Cly-PHA NPs exhibited a strong wound healing effect by eradicating bacterial colonies from the wound site (Figure 4B). Furthermore, they reduced the counts of mononuclear inflammatory cells and helped achieve an appearance similar to that of normal skin and healthy epidermal cells. Thus, NPs loaded with clindamycin can help eradicate bacterial colonies from the wound site and facilitate wound healing.

## 5. Conclusions

This study explored the potential use of PHA as a delivery carrier for antibiotics in the treatment of MRSA-infected cutaneous wounds. Cly-PHA NPs released clindamycin over a period of 2 days and demonstrated potent antibacterial effects against MRSA. Furthermore, the topical application of Cly-PHA NPs to MRSA-infected cutaneous wounds effectively eradicated the bacteria, leading to accelerated infected wound healing. These findings suggest that PHA is a promising material as a nanocarrier for antibiotics to treat MRSA-infected cutaneous wounds.

## Figures and Tables

**Table 1:** Characteristics of polyhydroxyalkanoate nanoparticles and clindamycin-loaded polyhydroxyalkanoates nanoparticles (PHA NPs and Cly-PHA NPs, respectively), including their size, polydispersity index (PDI), zeta potential, drug loading (DL%), and encapsulation efficiency (EE%).

Sample	Size (nm)	PDI	Zeta Potential (mV)	DL (%)	EE (%)
PHA NPs	180.9 ± 27.4	0.117 ± 0.010	−12.3 ± 0.4	----	--------
Cly-PHA NPs	216.2 ± 38.9	0.093 ± 0.030	−11.6 ± 0.5	6.76 ± 0.19%	74.06 ± 2.41%

Results are presented as means ± standard deviations (*n* = 3).

## Data Availability

The original contributions presented in the study are included in the article, and further inquiries can be directed to the corresponding author.
